# Prison Population Reductions and COVID-19: A Latent Profile Analysis Synthesizing Recent Evidence From the Texas State Prison System

**DOI:** 10.1007/s11524-020-00504-z

**Published:** 2020-12-18

**Authors:** Noel Vest, Oshea Johnson, Kathryn Nowotny, Lauren Brinkley-Rubinstein

**Affiliations:** 1 Department of Psychiatry and Behavioral Sciences, Stanford University School of Medicine, Palo Alto, CA 94304 USA; 2 Department of Sociology, University of Miami, Coral Gables, FL USA; 3 Department of Social Medicine, Center for Health Equity Research, University of North Carolina, Chapel Hill, NC USA

## Abstract

People in prison are particularly vulnerable to infectious disease due to close living conditions and the lack of protective equipment. As a result, public health professionals and prison administrators seek information to guide best practices and policy recommendations during the COVID-19 pandemic. Using latent profile analysis, we sought to characterize Texas prisons on levels of COVID-19 cases and deaths among incarcerated residents, and COVID-19 cases among prison staff. This observational study was a secondary data analysis of publicly available data from the Texas Department of Criminal Justice (TBDJ) collected from March 1, 2020, until July 24, 2020. This project was completed in collaboration with the COVID Prison Project. We identified relevant profiles from the data: a low-outbreak profile, a high-outbreak profile, and a high-death profile. Additionally, current prison population and level of employee staffing predicted membership in the high-outbreak and high-death profiles when compared with the low-outbreak profile. Housing persons at 85% of prison capacity was associated with lower risk of COVID-19 infection and death. Implementing this 85% standard as an absolute minimum should be prioritized at prisons across the USA.

**Supplementary Information:**

The online version contains supplementary material available at 10.1007/s11524-020-00504-z.

## Introduction

Due to their close living conditions and limited opportunity for physical distancing, people in prisons are extremely vulnerable to COVID-19 infection [1]. As a result, prisons have become hotspots for recent COVID-19 outbreaks [2]. There has been a 21.4% increase in COVID-19 cases in prisons from July 13, 2020, to July 26, 2020, such that persons incarcerated are infected at nearly 4 times the rate of the general public, and prison staff is infected at two and half times the rate of the general public [3]. Despite the surge in COVID-19 cases, little is currently known about what factors are responsible for increased rates of prison-level cases and deaths. A better understanding would provide prison administrators, researchers, and healthcare professionals with valuable information for public health policy and planning as it relates to COVID-19 infections in state prisons.

We use latent profile analysis (LPA) to provide data-driven patterns of the COVID-19 outbreak in the Texas Department of Criminal Justice (TDCJ), the largest state prison system. TDCJ has the highest level of COVID-19 cases and deaths in the nation and reports active cases at 97% of the prison facilities [3]. TDCJ provides a unique opportunity to examine data patterns because all residents and staff have been tested for COVID-19 [4, 5].

## Method

We used publicly available data from TDCJ and COVID Prison Project [4] collected since the beginning of the pandemic up to July 24, 2020. The primary outcome was a latent profile of Texas prisons based on their levels of incarcerated resident COVID-19 cases, incarcerated resident COVID-19 deaths, and staff COVID-19 cases. Secondary outcomes included prison-level predictors of latent profile membership (population, capacity, age of the prison, and staff levels^1^). We excluded three prison facilities because they were identified as holding facilities for individuals that had recently violated parole and did not report COVID-19 data. Our final sample included 103 Texas prison facilities reporting ranges from 0 to 791 current COVID-19 inmate cases, 0 to 12 inmate deaths, and 0 to 124 staff cases.

### Data Analysis

**Fig. 1:**
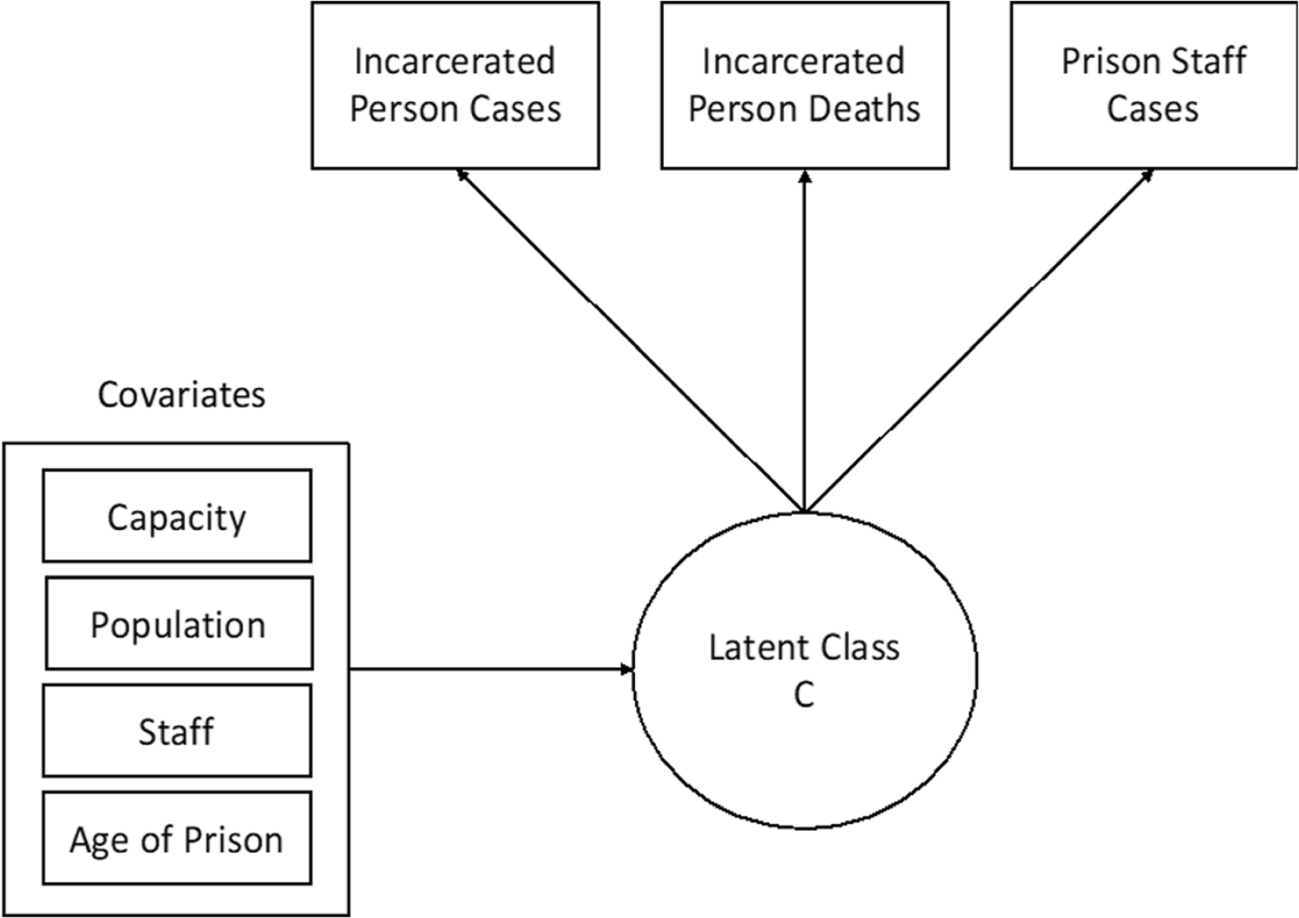
Statistical diagram of the full latent profile model with included covariates

We analyzed the data using MPlus version 8.3 [6]. First, LPA models were evaluated to determine the profile structure. We used LPA to group for patterns in the data based on three continuous observed indicators: (1) reported COVID-19 cases among incarcerated individuals, (2) reported COVID-19 deaths among incarcerated individuals, and (3) reported COVID-19 cases among prison staff (Fig. 1). We planned to include staff deaths, but the relatively low level of staff deaths created convergence problems.

We estimated 1–5 latent profile solutions. Model selection was based on standard fit statistics (BIC, entropy scores, and LRT scores) [7, 8]. Once the latent profiles were identified, we examined the association between covariates and profiles using a model-based multinomial logistic regression [9]. The three-step method was preferred because it produces more stable and less biased estimates with small sample sizes (e.g., 100–200) [10]. Each of the predictor covariates was entered into the model separately. For all of our logistic models, we used profile 1 as the referent profile. We had no missing data.

### Ethics

Because the data was publicly available, it did not require approval from the Stanford University Institutional Review Board.

## Results

There were 11,799 confirmed COVID-19 cases within TDCJ among incarcerated residents, 104 presumed COVID-19 deaths among incarcerated residents, 2497 confirmed cases among prison staff, and 12 deaths among prison staff. The entire resident population of the prisons included in the analyses was 130,610, and the entire staff population was 37,201.

**Fig. 2:**
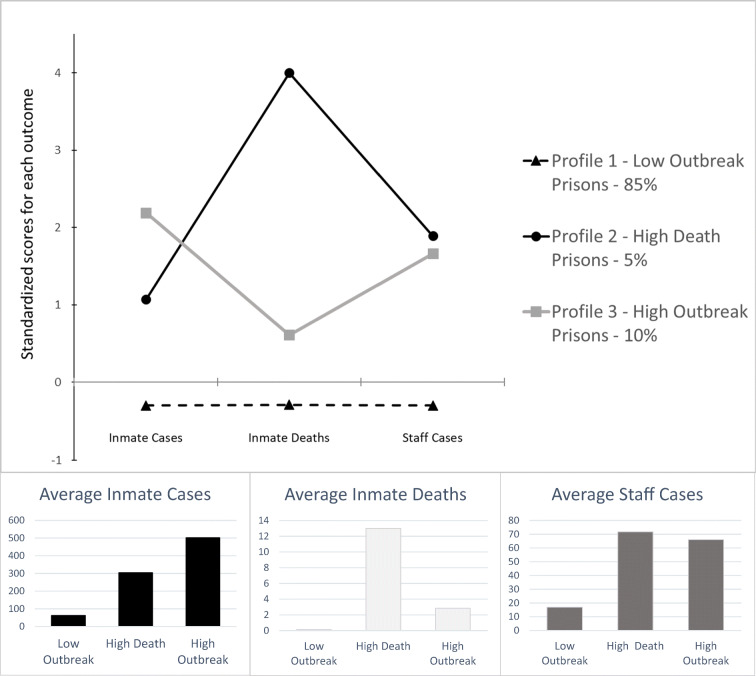
Standardized and actual mean values for inmate cases, inmate deaths, and staff cases for latent profiles 1–3

We identified the three-profile solution as the most parsimonious (BIC 2595.08, entropy .99, non-significant LMR). Profile 1 (88 out of 103 prisons—dashed black line in Fig. 2), “low-outbreak” facilities, was characterized by prisons with a low number of incarcerated resident cases, a low number of incarcerated resident deaths, and a low number of prison staff cases. Profile 2 (5 out of 105 prisons—black line in Fig. 2), “high-death” facilities, was characterized by prisons with a moderate number of incarcerated resident cases, a very high level of incarcerated resident deaths, and a high level of prison staff cases. Profile 3 (10 out of 103 prisons—gray line in Fig. 2), “high-outbreak” facilities, was characterized by prisons with a very high level of incarcerated resident cases, a moderate level of incarcerated resident deaths, and a high level of staff cases. In Supplement 1, we offer a listing of the prisons in each profile.

The effects of covariates on profile membership were analyzed for differences from profile 1 (low outbreak; Table 1). Current prison population significantly predicted membership in the high-outbreak and high-death profiles when compared with the low-outbreak profile. Low-outbreak prisons were at 85% of capacity, while the high-death and high-outbreak profiles were at 94% and 102% capacity, respectively. Current number of employees significantly predicted membership in the high-outbreak and high-death profiles compared with the low-outbreak profile. We found no statistical differences among profiles in age of operation for a prison.^2^

**Table 1:** Prison characteristic and risk covariates

Variable–Mean/% (SE), *p* value	Profile 1 (referent)	Profile 2	Profile 3
Current facility capacity	1238.28 (77.34)	2016.60 (490.96), *p* = .06	2568.11 (341.63), *p* < .01
Current facility population	1077.77 (75.65)	1934.60 (472.73), *p* = .03	2603.90 (299.52), *p* < .01
Population to capacity ratio^a^	85%	94%	102%
Employees	315.68 (18.08)	571.20 (149.26), *p* = .03	656.50 (73.91), *p* < .01
Employee to population ratio^a^	1 to 3.4	1 to 3.4	1 to 4.0
Years in operation	37.98 (3.18)	52.60 (21.22), *p* = .25	36.70 (3.44), *p* = .78

*P* values less than .05 specify that the odds ratio for the profile indicated a significant difference from the referent group. ^a^denotes that this ratio was included for explanatory purposes only and was not included in the analysis of statistical differences between profiles. Profile 1 = low outbreak; profile 2 = high death; profile 3 = high outbreak

^a^denotes that this ratio was included for explanatory purposes only and was not included in the analysis of statistical differences between profiles. Profile 1 = low outbreak; profile 2 = high death; profile 3 = high outbreak

## Discussion

This is the first study to examine COVID-19 cases in a statewide prison system. We found that the majority of prisons in Texas were characterized by low levels of COVID-19 outbreaks among staff and incarcerated residents. Additionally, the level of overcrowding in the low-outbreak prisons was moderate with a current population to capacity ratio of 85%. This suggests that the benchmark for prisons to effectively reduce COVID-19 infections should be set to under 85% of capacity. Importantly, this 85% standard should be implemented as an absolute minimum rate, with further reductions for high-risk geriatric and medical facilities.

Our findings suggest that more than half of the total number of COVID-19 deaths in Texas were attributed to five prisons (65 out of 103 deaths), and nearly half of the total cases of COVID-19 were attributed to 10 prisons (5000 out of 11,799 people—please see Supplement 1 for the full list of prisons in each profile). This suggests that there are COVID-19 prison hotspots, which may be connected to the overcrowding issue, understaffing, or other common characteristics that facilities share, such as resident demographics. For example, two prisons in the “high-death” profile are geriatric facilities (e.g., Duncan and Pack). Importantly, differences in population to capacity rates between the high-death and high-outbreak prisons may be due to relevant individual characteristics such as resident age or the prevalence of comorbid medical conditions.

In this inherently overcrowded environment, we suggest prisons should continue to drastically reduce their prison populations through decarceration efforts as a best practice to mitigate harms [11]. This is especially true for those over 55 years of age [12] since there is a growing need for gerontological knowledge and skills in prisons to help mitigate the growing infection and death rates occurring among the older incarcerated population [13]. Lastly, age of the prison was not predictive of profile membership which suggests that building new prisons may not mitigate the public health crisis of COVID-19 infections in the prison environment.

### Limitations

This study has several limitations. First, we did not account or control for other potentially important prison-level or person-level characteristics. Future studies will be needed to determine the impact of prison specific variables such as average cell size, ventilation, visitation policies, telemedicine, transfer rate, security level, number of infirmary beds, and protective equipment availability and mandates. As well, the impact of person-level variables such as sex and preexisting health conditions warrants further evaluation. Second, the current study only examines Texas prison facilities and may not be generalizable to other state prison systems. However, we feel that because hundreds of thousands of lives may be at stake in prison systems across the world, these findings should not only inform policy on the prison system in Texas but globally. Third, data is updated daily; therefore, the number of tests, cases, and deaths of incarcerated individuals and staff will change as time progresses, and our results only capture data up to July 24, 2020.

## Conclusions

We implemented a unique data-driven statistical technique to divide prisons into clusters based upon reported levels of infections and deaths at the state level. These findings should inform researchers, prison administrators, lawmakers, public health officials, and other professionals interested in reducing the impact of COVID-19 in our nation’s prisons. Importantly, housing people incarcerated at 85% of facility capacity may minimize the rate of infection and death in state prisons.

## Supplementary Information

ESM 1 (DOCX 20 kb)

## Footnotes

1 These prison-level predictors were specifically chosen because they were publically available for each prison.

2 We considered age of the prison to be a proxy for “architectural design” of the prison, which has been shown to influence other infectious diseases. Improving the common elements of architectural design (ventilation and sharing of cells) may lead to improvements without expanding the number of prisons.
