# Supplementary material for: Prison Population Reductions and COVID-19: A Latent Profile Analysis Synthesizing Recent Evidence From the Texas State Prison System
Source: J Urban Health. 2020 Dec 18;98(1):53–8. doi: 10.1007/s11524-020-00504-z (PMC7747775; doi:10.1007/s11524-020-00504-z)
Supplement: Supplementary file 1 — (DOCX 20 kb) [file 11524_2020_504_MOESM1_ESM.docx]

Supplemental File 1.

The three facilities excluded from the analyses were Baten, Kegan, and Rudd.

Profile 1 Prisons – Low Outbreak

Low Inmate Cases – Low Inmate Deaths – Low Staff Cases

Allred

Bell

Boyd

Bradshaw

Bridgeport

Briscoe

Byrd

Clemens

Cole

Connally

Cotulla

Crain

Dalhart

Daniel

Darrington

Diboll

East Texas

Eastham

Estes

Ferguson

Formby

Fort Stockton

Garza East

Garza West

Gist

Glossbrenner

Goodman

Goree

Gurney

Halbert

Hamilton

Havins

Henley

Hightower

Hilltop

Hobby

Hodge

Holliday

Hospital Galveston

Hughes

Huntsville

Hutchins

Jester I

Jester IV

Johnston

Jordan

Kyle

LeBlanc

Lewis

Lindsey

Lockhart

Lopez

Luther

Lychner

Lynaugh

Marlin

McConnell

Middleton

Montford/West Texas Hospital

Moore, B.

Moore, C.

Mountain View

Murray

Neal

Ney

Plane/Santa Maria Baby Bonding

Powledge

Ramsey

Roach

Robertson

San Saba

Sanchez

Sayle

Scott

Segovia

Skyview

Smith

Stevenson

Stringfellow

Torres

Travis County

Tulia

Vance

Wallace/San Angelo Work Camp

Wheeler

Willacy County

Woodman

Young

Profile 2 Prisons – High Death

Moderate Inmate Cases – Extremely High Inmate Deaths – High Staff Cases

Duncan

Estelle

Pack

Telford

Wynne

Profile 3 Prisons – High Outbreak

Extremely High Inmate Cases – Moderate Inmate Deaths – High Staff Cases

Beto

Clements

Coffield

Dominguez

Ellis

Jester III

Michael

Polunsky

Stiles
